# Kainate Receptor Auxiliary Subunit NETO2-Related Cued Fear Conditioning Impairments Associate with Defects in Amygdala Development and Excitability

**DOI:** 10.1523/ENEURO.0541-19.2020

**Published:** 2020-08-27

**Authors:** Marie Mennesson, Ester Orav, Adrien Gigliotta, Natalia Kulesskaya, Suvi Saarnio, Anna Kirjavainen, Sebnem Kesaf, Frederike Winkel, Maria Llach Pou, Juzoh Umemori, Vootele Voikar, Victoria Risbrough, Juha Partanen, Eero Castrén, Sari E. Lauri, Iiris Hovatta

**Affiliations:** 1 Department of Psychology and Logopedics, Medicum, University of Helsinki, Helsinki 00290, Finland; 2 Molecular and Integrative Biosciences Research Program, University of Helsinki, Helsinki 00790, Finland; 3 Neuroscience Center, Helsinki Institute of Life Science HiLIFE, University of Helsinki, Helsinki 00290, Finland; 4 SleepWell Research Program, Faculty of Medicine, University of Helsinki, Helsinki 00290, Finland; 5 Center of Excellence for Stress and Mental Health, Veterans Affairs, La Jolla, CA 92093; 6 Department of Psychiatry, University of California, La Jolla, CA 92093

**Keywords:** amygdala, excitability, fear conditioning, immunohistochemistry, interneuron, knock-out mouse

## Abstract

NETO2 is an auxiliary subunit for kainate-type glutamate receptors that mediate normal cued fear expression and extinction. Since the amygdala is critical for these functions, we asked whether *Neto2*^−/−^ mice have compromised amygdala function. We measured the abundance of molecular markers of neuronal maturation and plasticity, parvalbumin-positive (PV^+^), perineuronal net-positive (PNN^+^), and double positive (PV^+^PNN^+^) cells in the *Neto2*^−/− ^amygdala. We found that *Neto2*^−/−^ adult, but not postnatal day (P)23, mice had 7.5% reduction in the fraction of PV^+^PNN^+^ cells within the total PNN^+^ population, and 23.1% reduction in PV staining intensity compared with *Neto2*^+/+^ mice, suggesting that PV interneurons in the adult *Neto2*^−/−^ amygdala remain in an immature state. An immature PV inhibitory network would be predicted to lead to stronger amygdalar excitation. In the amygdala of adult *Neto2*^−/−^ mice, we identified increased glutamatergic and reduced GABAergic transmission using whole-cell patch-clamp recordings. This was accompanied by increased spine density of thin dendrites in the basal amygdala (BA) compared with *Neto2*^+/+^ mice, indicating stronger glutamatergic synapses. Moreover, after fear acquisition *Neto2*^−/−^ mice had a higher number of c-Fos-positive cells than *Neto2*^+/+^ mice in the lateral amygdala (LA), BA, and central amygdala (CE). Altogether, our findings indicate that *Neto2* is involved in the maturation of the amygdala PV interneuron network. Our data suggest that this defect, together with other processes influencing amygdala principal neurons, contribute to increased amygdalar excitability, higher fear expression, and delayed extinction in cued fear conditioning, phenotypes that are common in fear-related disorders, including the posttraumatic stress disorder (PTSD).

## Visual Abstract

**Figure:**
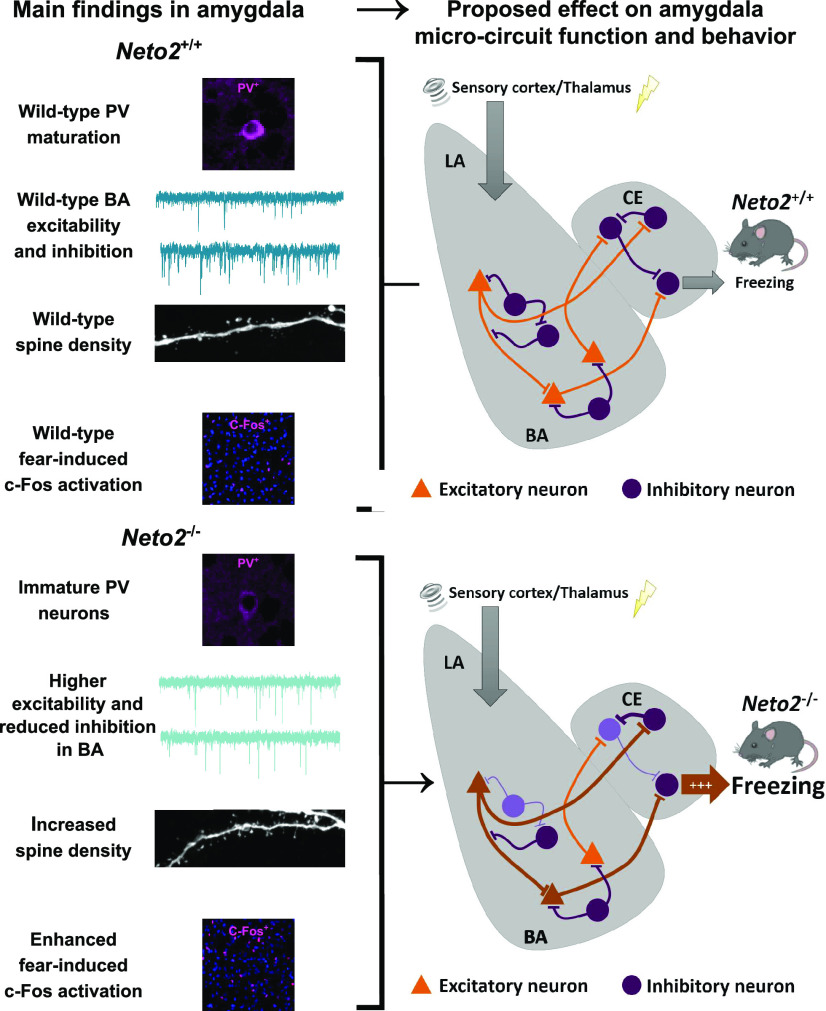

## Significance Statement

NETO2 is required for normal fear expression and extinction in cued fear conditioning, but the underlying mechanisms remain unknown. Since amygdala is a central brain region regulating fear responses, we investigated its maturation and function in *Neto2*^−/−^ and *Neto2*^+/+^ mice. *Neto2*^−/−^ mice had stronger fear expression and slower extinction than *Neto2*^+/+^ mice, associated with amygdala hyperexcitability. We propose that defects in the *Neto2*^−/−^ mice involving amygdala PV-interneuron network configuration and amygdala excitation and inhibition imbalance through multiple mechanisms contribute to the fear phenotype of these mice. These findings could inform novel targets for fear-related disorders, such as phobias and posttraumatic stress disorder (PTSD).

## Introduction

NETO2 is an auxiliary subunit of kainate-type ionotropic glutamate receptors (KARs), and it thereby modulates biophysical properties and agonist affinity of KARs (Zhang et al., 2009; Wyeth et al., 2017). In addition, NETO2 also interacts with K-Cl co-transporter 2 (KCC2), and together with KARs influence its expression in the hippocampal neurons (Ivakine et al., 2013; Pressey et al., 2017). Both male and female *Neto2*^−/−^ mice have normal innate anxiety-like behavior, but greater fear expression and delayed extinction in cued fear conditioning compared with *Neto2*^+/+^ mice, resembling fear phenotypes associated with posttraumatic stress disorder (PTSD; Mennesson et al., 2019). Furthermore, the synaptic KAR abundance of *Neto2*^−/−^ mice is significantly reduced in fear-related brain regions, including the amygdala, medial prefrontal cortex (mPFC), and ventral hippocampus (vHpc; Mennesson et al., 2019).

The amygdala is a group of heterogeneous nuclei central to fear learning, memory consolidation, and fear expression. Its main subnuclei are the lateral (LA), basal (BA), and central (CE) amygdala (Herry and Johansen, 2014; Tovote et al., 2015). Fear-inducing stimuli are processed in the LA, where both cortical and thalamic inputs project onto glutamatergic principal neurons (Ehrlich et al., 2009; Herry and Johansen, 2014), mediating neural plasticity and fear learning (Johansen et al., 2010, 2011). LA neurons project to the BA, and both LA and BA neurons project directly and indirectly to the CE (Herry et al., 2010). Fear expression is regulated by multiple circuits involving the BA, CE, mPFC, and periaqueductal gray (Herry and Johansen, 2014). At the cellular level, parvalbumin (PV)-expressing interneurons tightly regulate the activity of the glutamatergic principal neurons within the amygdala (Wolff et al., 2014). This ongoing synaptic inhibition controls whether inputs to the principal neurons undergo synaptic plasticity, allowing the formation of new fear memories (Ehrlich et al., 2009). Both presynaptic and postsynaptic KARs contribute to induction of long-term potentiation (LTP) in the LA and BA (Li et al., 2001; Ko et al., 2005; Shin et al., 2010; Cho et al., 2011). Mice lacking the *Grik2* gene, encoding the GLUK2 subunit of KAR, have reduced cued and contextual memory recall after fear conditioning indicating impaired fear memory consolidation (Ko et al., 2005). Thus, regulation of synaptic plasticity in the amygdala via PV interneurons and KARs may modulate fear-related behaviors.

PV interneuron maturation and plasticity are influenced by perineuronal nets (PNNs; Beurdeley et al., 2012; Wang and Fawcett, 2012). PNNs are specialized extracellular matrix structures that stabilize synaptic connections onto neurons they surround (Wang and Fawcett, 2012) and are considered as a mark of synaptic stability and consolidated memory (Lorenzo Bozzelli et al., 2018). They prevent LTP at excitatory synapses in the hippocampus (Carstens et al., 2016) and protect fear memories from erasure during extinction in the amygdala (Gogolla et al., 2009; Gunduz-Cinar et al., 2019). Consequently, the number of PV cells surrounded by PNN is used as a marker of neuronal plasticity (Nowicka et al., 2009; Umemori et al., 2018). Within the PV-positive (PV^+^) cells, the intensity of PV immunoreactivity increases during development (Donato et al., 2013; Umemori et al., 2015) and is suggested to be an indicator of neuronal maturity (Umemori et al., 2018).

NETO2 regulates interneuron function in the developing hippocampus (Wyeth et al., 2017), where it is also developmentally downregulated (Orav et al., 2017). Thus, we asked whether *Neto2* influences interneuron maturation in the amygdala with consequences on fear memory. We studied the PV staining intensity and the number of PV cells surrounded by PNN (PV^+^PNN^+^) as markers of amygdala PV interneuron network maturity in juvenile postnatal day (P)23 mice and in ∼12-week-old adult mice. Following our finding that in adult, but not in the P23, mice, the fraction of PV^+^PNN^+^ cells relative to the total PNN^+^ population was reduced and that the PV staining intensity was lower in *Neto2*^−/−^ compared with *Neto2*^+/+^ mice, we conducted electrophysiological recordings in the amygdala neurons and measured amygdalar activation after fear acquisition and extinction using c-Fos immediate early gene mapping. Our results indicate that NETO2 deficiency perturbs excitability of the BA, and that defects in these functions may contribute to the higher fear expression and delayed extinction of *Neto2*^−/−^ mice.

## Materials and Methods

### Animals

*Neto2*^−/−^ mice were created as previously described (Tang et al., 2011). Mice were maintained on a 12/12 h light/dark cycle (lights on at 6 A.M.) in individually ventilated cages (Mouse IVC Green Line, overall dimensions 391 × 199 × 160 mm, floor area 501 cm^2^; Tecniplast) with free access to water and food. Mice were single housed in IVCs one week before and during the test period to avoid possible effects of social hierarchy on behavior. All animal procedures were performed in accordance with the University of Helsinki animal care committee’s regulations. Experimenter was blind to the genotype during all experiments.

### Cued fear conditioning and extinction

Male and female mice were on average 12 weeks old (range 10–15 weeks) during fear conditioning. Testing was performed between 8 A.M. and 3 P.M. to avoid possible effects of the corticosterone secretion peak at the beginning of the dark phase (6 P.M.). Animals were brought in the testing room 30 min before the beginning of the test session. Cued fear conditioning and extinction were performed as previously described (Fitzgerald et al., 2015). Fear acquisition was performed in context A, a square transparent chamber (23 × 23 × 35 cm) with a grid floor delivering the foot shock [unconditioned stimulus (US), 0.6 mA], which was cleaned with ethanol between animals. The chamber was inside a white, constantly illuminated (100-lux) box, which contained loudspeakers delivering constant background noise (68 dB) and the sound cue [conditioned stimulus (CS), sine waves (pure sound) of 10 kHz, 76 dB, pulsed at 5 Hz]. During the acquisition on the first day, a sound cue (CS, 30 s) followed by a foot shock (US, 2 s) was delivered three times [intertrial interval (ITI) 30 s after a 120-s habituation period]. Mice remained in the chamber for 160 s after the last CS–US pairing. On the second day, fear extinction was conducted by presenting 20 CS without foot shocks (20 × 30 s after a 180-s habituation period, ITI 5 s) in context B, a square black chamber (23 × 23 × 35 cm) wherein the grid floor was covered, which was cleaned between animals with water containing 1% vanilla extract. Experiments were conducted using a computer-controlled fear conditioning system (TSE), and percent time freezing was measured automatically by an infrared detection system. Percent time freezing during extinction was analyzed as an average of four CS presentations. For the c-Fos staining, two groups of adult mice were killed exactly 90 min after the end of the acquisition [wild type (WT) *n* = 8, knock-out (KO) *n* = 7] or the extinction (WT *n* = 10, KO *n* = 10; Fig. 6*A*).

### Immunohistochemistry (IHC)

Mice were injected with a lethal dose (600 mg/kg) of pentobarbital (Mebunat Vet 60 mg/ml, Orion Pharma) and transcardially perfused with PBS (137 mm NaCl, 2.7 mm KCl, 10 mm Na_2_HPO_4_ 2H_2_O, and 1.8 mm KH_2_PO_4_; pH 7.4), followed by 4% paraformaldehyde (PFA) in PBS, both at 37°C. Brains were post fixed 2–4 d in PFA at 4°C, dehydrated, and embedded in paraffin blocks and sectioned (10 µm) using a Leica RM2255 microtome (Leica Biosystems). Sections were deparaffinized and rehydrated before heat-induced antigen retrieval (0.01 m sodium citrate, Tween 0.05%, pH 6.0, heated in microwave oven for 15 min). Sections were washed using PBS + 0.2% Triton X-100 (PBST) for 3 × 5 min and incubated 60 min with blocking solution containing 10% normal goat serum (NGS; 005-000-121, Jackson ImmunoResearch) in PBST for c-Fos staining, and 3% BSA (001-000-162, Jackson ImmunoResearch) + 10% NGS in PBST for PV and PNN staining. Sections were then incubated 24–48 h with rabbit anti-c-Fos (1:500, sc-52, Santa Cruz Biotechnology) primary antibody in 5% NGS in PBST, or with mouse anti-PV (1:5000, PV235, Swant Swiss antibodies) primary antibody, and Wisteria floribunda agglutinin (WFA; detects chondroitin sulfate chains enriched in PNNs) conjugated with biocytin (1:400, L1516, Sigma-Aldrich) in 0.2% BSA + 10% NGS in PBST. Sections were washed 3 × 5 min with PBST and, depending on the primary antibody, incubated for 1–2 h with goat anti-rabbit secondary antibody conjugated with Alexa Fluor 568 (A-11011, ThermoFisher Scientific) or goat anti-mouse secondary antibody conjugated with Alexa Fluor 488 (A-11029, ThermoFisher). WFA-biocytin was detected using streptavidin conjugated with Alexa Fluor 568 (S11226, ThermoFisher Scientific) in PBST. Sections were rinsed 3 × 5 min with PBS and mounted using VECTASHIELD Antifade Mounting Medium with DAPI (VECTOR Laboratories).

### Imaging and IHC data analysis

Brain sections were imaged using a Zeiss fluorescent microscope (Axio Imager 2, Carl Zeiss) and processed and exported with the Zeiss Zen Lite Software (Carl Zeiss). For c-Fos and PV/WFA staining, images were analyzed with a semi-automated macro in ImageJ (manual settings: region of interest selection, counting of PNN/PV-positive cells, PV staining intensity) and without adjusting for exposure time and background before the analysis. PV intensity was calculated from the integrated density (area*mean gray value) of PV staining normalized by the intensity of the background near the PV cell soma. A cell was considered c-Fos^+^ if it co-localized >75% with nuclear DAPI staining. c-Fos intensity was calculated using the area (µm^2^)*mean gray value of each positive c-Fos cell (integrated density) and normalized by the integrated density of the background from the selected regions of interest.

### Electrophysiological recordings

We prepared 350-µm coronal acute slices from brains of 12-week-old *Neto2*^+/+^ and *Neto2*^−/−^ littermate mice. During the dissection and slicing, the brain was kept in an ice-cold solution containing the following: 87 mm NaCl, 2.5 mm KCl, 7 mm MgCl_2_, 1.25 mm NaH_2_PO_4_, 0.5 mm CaCl_2_, 25 mm NaHCO_3_, 50 mm D-sucrose, and 25 mm D-glucose (all from Sigma-Aldrich) and saturated with 95% O_2_ and 5% CO_2_. Immediately after cutting the slices were transferred to ACSF containing the following: 124 mm NaCl, 3 mm KCl, 1.25 mm NaH_2_PO_4_, 1 mm MgSO_4_, 26 mm NaHCO_3_, 15 mm D-glucose, 2 mm CaCl_2_; 5% CO_2_/95% O_2_ and incubated 30 min at +35°C and 30 min to 5 h at room temperature before use.

For recordings, the slice was placed in a submerged recording chamber and continuously perfused with ACSF at +30°C. Whole-cell voltage-clamp recordings were made from LA and BA under visual guidance using patch electrodes with resistance of 4–6 MΩ. Uncompensated series resistance (Rs) was monitored by measuring the peak amplitude of the current response to a 5-mV step. Only experiments where Rs < 30 MΩ, and with < 20% change in Rs during the experiment, were included in the analysis. In addition, cells with very low capacitance (<35 pF) were considered as GABAergic interneurons and excluded from the analysis.

For recording of spontaneous synaptic currents, patch pipette was filled with a solution containing the following: 135 mm K-gluconate, 10 mm HEPES, 2 mm KCl, 2 mm Ca(OH)_2_, 5 mm EGTA, 4 mm Mg-ATP, and 0.5 mm Na-GTP; 285 mOsm and pH was adjusted to 7.2 with NaOH (all from Sigma-Aldrich). Spontaneous glutamatergic events (sEPSCs) were recorded at a holding potential of −70 mV, after which the cell was voltage clamped at 0 mV to record spontaneous GABAergic currents (sIPSCs). mEPSC recordings were made with an intracellular solution containing the following: 130 mm CsMeSO_4_, 10 mm HEPES, 0.5 mm EGTA, 4  mm Mg-ATP, 0.3 mm Na-GTP, 5 mm QX-314, and 8 mm NaCl; 285 mOsm and pH was adjusted to 7.2 with CsOH (all from Sigma-Aldrich). mEPSCs were recorded in the presence of 1 μm TTX (Abcam), 100 μm Picrotoxin (Abcam), and 50 μm AP5 (HelloBio) at a holding potential of −70 mV. mIPSC recordings were made with an intracellular solution containing the following: 145 mm CsCl, 10 mm HEPES, 5 mm EGTA, 2 mm MgCl_2_, 2 mm CaCl_2_, 4 mm Mg-ATP, and 0.33 mm Na-GTP; 278 mOsm and pH was adjusted to 7.2 with CsOH (all from Sigma-Aldrich). mIPSCs were recorded in the presence of 50 μm AP5 (HelloBio), 25 μm GYKI53655 (Tocris), 1 μm TTX (Abcam) at a holding potential of −70 mV. Biocytin (0.3%; Sigma-Aldrich) was added to both intracellular solutions to later visualize and analyze cell morphology and to confirm the location of the recorded neuron. Data were collected using WinLTP software (WinLTP Ltd) and analyzed offline using the MiniAnalysis 6.0.3 program (Synaptosoft Inc.). The threshold for detection of both inward (sEPSC, mEPSC, mIPSC) and outward (sIPSC) events was three times the baseline noise level, and all detected events were verified visually. The experimenter was blind to the mouse genotype throughout the experiment until statistical analysis.

### Biocytin staining and spine analysis

After filling the recorded neurons with biocytin, the slices were fixed with 4% PFA in PBS. The fixed slices were washed first with PBS, then with PBS + 0.3% Triton X-100 solution (PBST) and blocked in 0.4% NGS in PBST. Biocytin was visualized using streptavidin conjugated with Alexa Fluor 488 (Invitrogen). Slices were mounted using VECTASHIELD Hard Set Antifade Mounting Medium with DAPI (VECTOR Laboratories). First branch dendrites of the neurons were imaged using Zeiss LSM 700 Confocal microscope (63× objective, 2.6 zoom, interval of stacked images 0.25 µm, Axio Imager 2, Carl Zeiss), and the images were processed and exported with Zeiss Black Software (Carl Zeiss). Spine density was measured *post hoc* from primary branches and analyzed using NeuronStudio software (Computational Neurobiology and Imaging Center, Mount Sinai School of Medicine, NY). Spines were classified as mushroom, thin, or stubby using NeuronStudio spine classifier settings. Briefly, if no neck was present, spine was classified as stubby, and in the case a neck was present, the head size diameter threshold was used to distinguish between thin and mushroom spines (<0.35 µm for thin and >0.35 µm for mushroom). Length and thickness of dendrites were measured using NeuronStudio software neurites and measurement tools, respectively. The experimenter was blind to the mouse genotype throughout the experiment until statistical analysis.

### Statistical analysis

*Neto2* ablation influences fear expression and extinction in both male and female mice (Mennesson et al., 2019), and therefore, we included both sexes in this study. Because of absence of sex effects in the behavioral data, we analyzed males and females jointly in all experiments. Dependent variables were checked for normal distribution (Shapiro–Wilk test, *p* > 0.05) and were analyzed using mixed ANOVA, generalized estimating equation (GEE), or Kolmogorov–Smirnov tests (Table 1). GEE analysis was used to control for within-subject dependencies of individual data points collected from the same animal (e.g., neurons recorded from slices originating from the same mouse) or from the same cell/image (e.g., PV cell intensities measured from the same image), as previously described (Hanley et al., 2003; Laine et al., 2018). Statistical analyses were conducted using SPSS Statistics 24 (IBM), GraphPad Prism7 (GraphPad Software), or RStudio (RStudio Inc.).

**Table 1:** Statistical table

Figure	Data structure	Statistical test	Significance threshold (α)
1*B–E*	Normally/not normally distributed	Generalized estimating equation (GEE)	<0.05
2*A*,*B*	Not normally distributed	Two sample Kolmogorov–Smirnov test	<0.05
2*C–E*	Not normally distributed	Generalized estimating equation (GEE)	<0.05
3*A–D*	Normally/not normally distributed	Two sample Kolmogorov–Smirnov test	<0.05
		Generalized estimating equation (GEE)	<0.05
4*A*,*B*	Bimodally distributed	Two sample Kolmogorov–Smirnov test	<0.05
4*C–F*	Normally distributed	Generalized estimating equation (GEE)	<0.05
5*A*,*B*	Normally distributed	Two sample Kolmogorov–Smirnov test	<0.05
5*A–E*	Normally/not normally distributed	Generalized estimating equation (GEE)	<0.05
6*B*	Normally distributed	Mixed ANOVA (repeated measures)	<0.05
6*E–H*	Normally/not normally distributed	Generalized estimating equation (GEE)	<0.05

## Results

### PV interneuron state of the *Neto2*^−/−^ amygdala

**Figure 1.**
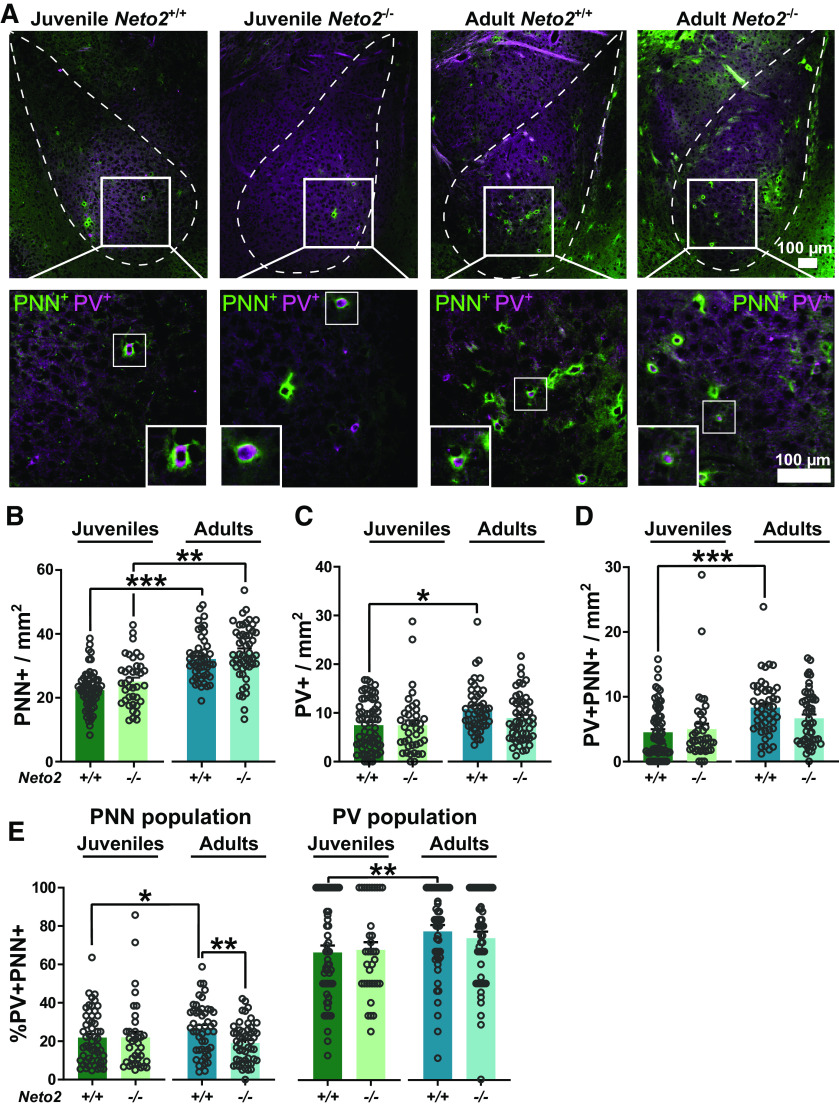
PV interneuron state of the *Neto2*^−/−^ amygdala. ***A***, Representative images of WFA (PNN) and PV staining in the amygdala of juvenile and adult mice. Examples of double positive cells are marked with a white square and corresponding close-ups are shown. Abundance of (***B***) PNN^+^, (***C***) PV^+^, and (***D***) PV^+^PNN^+^ cells in the amygdala of juvenile and adult mice. ***E***, Percentage of PV^+^PNN^+^ double positive cells in the total PNN^+^ cell population and total PV^+^ cell population. Each dot represents data from one image. Mean ± SEM is shown. Genotype effect calculated using GEE analysis; *p <* *0.05, **p <* *0.01, ***p <* *0.001.

*Neto2*^−/−^ mice have increased fear expression and delayed extinction in cued fear conditioning compared with the *Neto2*^+/+^ mice (Mennesson et al., 2019). Since the amygdala is the key brain region for processing of sensory information related to fear and anxiety, and in consolidation of fear memories, we asked whether NETO2 regulates development and function of the amygdala. We first investigated well-established molecular markers of maturity and plasticity involved in fear memory consolidation, the number of PNNs and PV-expressing interneurons (Gogolla et al., 2009; Donato et al., 2013, 2015). We concentrated on the LA and BA, the targets of subcortical projections from the acoustic thalamus. Since the number of PNNs increase during development, we studied two time points, P23 (juveniles) and ∼12 weeks (adults). The juvenile time point is of interest because P23 mice have stronger fear expression during cue retrieval (Pattwell et al., 2011) and faster fear extinction of cued fear memory than adults (Pattwell et al., 2012). We quantified the number of PNN^+^, PV^+^, and double positive PV^+^PNN^+^ cells (Fig. 1*A–D*) and calculated the percentage of PV^+^PNN^+^ cells relative to the total population of PNN^+^ or PV^+^ cells, as previously (Karpova et al., 2011; Ohira et al., 2013; Guirado et al., 2014; Fig. 1*E*). Overall, we found a larger number of PNN^+^ than PV^+^ cells, and only a subset of PNN^+^ cells were also PV^+^ in the LA/BA (Fig. 1*A*), as reported previously (Morikawa et al., 2017). As expected (Gogolla et al., 2009; Nowicka et al., 2009; Umemori et al., 2015), we detected a larger number of PNN^+^ (*p *=* *1.4E-11), PV^+^ (*p *=* *0.013), and PV^+^PNN^+^ (*p *=* *0.0001) cells in the adult compared with juvenile WT mice (Fig. 1*B–D*). However, in the *Neto2*^−/−^ mice, the abundance of PV^+^ and PV^+^PNN^+^ did not differ between juveniles and adults, and only the number of the PNN^+^ cells was significantly higher in adults than juveniles (PNN^+^ *p *=* *0.0016, PV^+^ *p *=* *0.29, PV^+^PNN^+^ *p *=* *0.12; Fig. 1*B–D*). Yet, the number of PNN^+^, PV^+^, and PV^+^PNN^+^ cells did not differ significantly between *Neto2*^−/−^ and *Neto2*^+/+^ mice in either juvenile or adult time points (Fig. 1*B–D*). However, there was a trend for reduced numbers of PV^+^PNN^+^ cells in adult *Neto2*^−/−^ compared with *Neto2*^+/+^ mice (PV^+^PNN^+^, *p *=* *0.054; Fig. 1*D*). Adult *Neto2*^+/+^ mice had more PV^+^PNN^+^ cells compared with juvenile *Neto2*^+/+^ mice relative to both PNN and PV cell populations (*p *=* *0.046 and *p *=* *0.004, respectively), while no differences were detected between ages in *Neto2*^−/−^ mice (Fig. 1*E*). *Neto2*^−/−^ adult, but not juvenile mice, had a significantly lower percentage of PV^+^PNN^+^ cells within the total PNN population compared with *Neto2*^+/+^ mice (*p *=* *0.003; Fig. 1*E*). Altogether, these results suggest defects in the maturation of PV interneurons in *Neto2*^−/−^ mice between P23 and adult ages.

**Figure 2.**
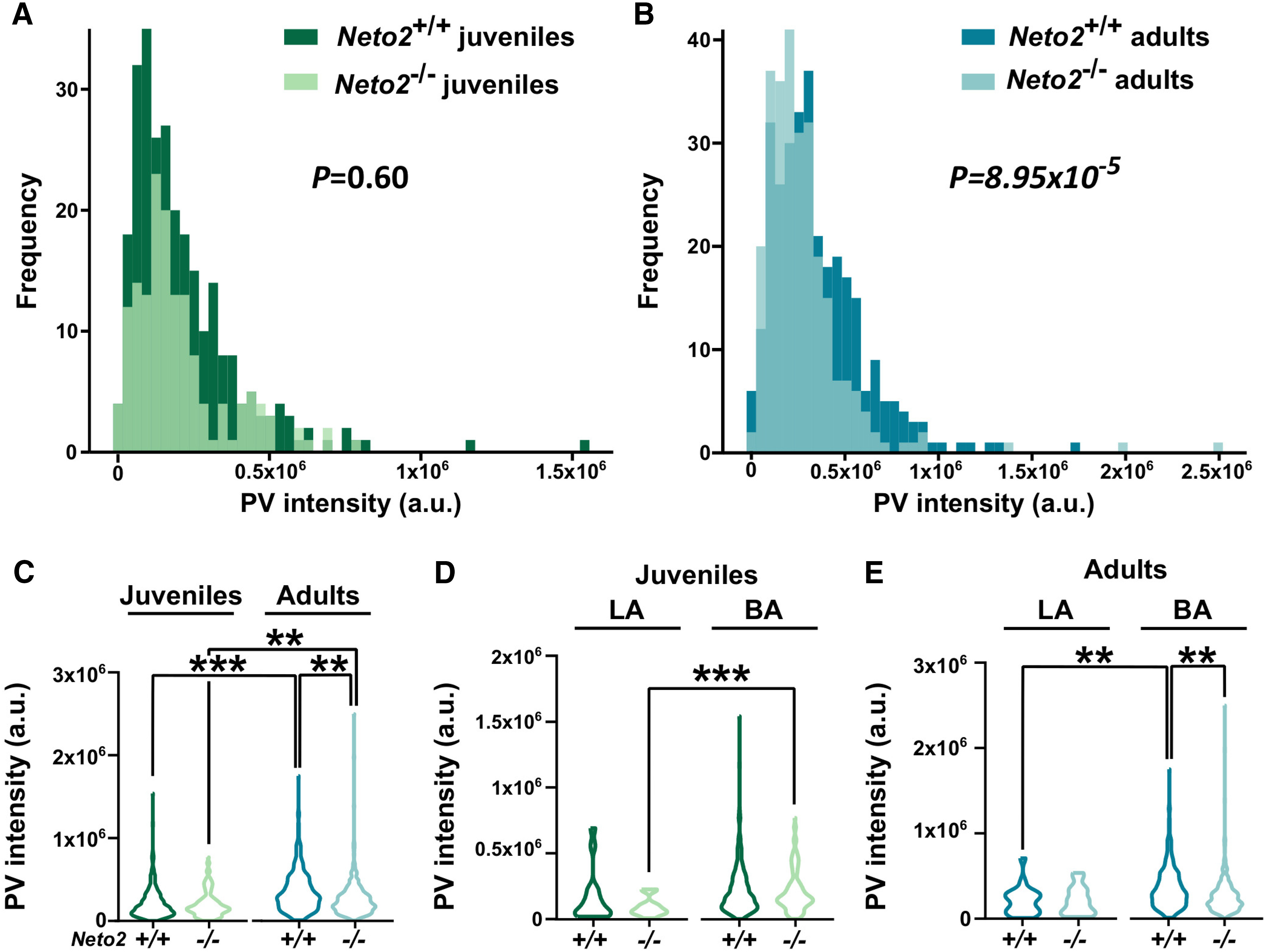
PV interneuron intensity is reduced in adult *Neto2*^−/−^ amygdala. PV intensity distribution in *Neto2*−/− and *Neto2*^+/+^ mice at (***A***) juvenile and (***B***) adult age. Mean PV intensity in the LA/BA (***C***) and in the LA and BA nuclei analyzed separately at (***D***) juvenile and (***E***) adult age. Mean ± SEM is shown. Genotype effect calculated using Kolmogorov–Smirnov (***A***, ***B***) or GEE (***C–E***) analysis; **p <* *0.01, ***p <* *0.001.

To further investigate the maturation of the PV inhibitory network within the LA/BA, we measured PV staining intensity (Fig. 2). Donato et al. (2013) have established that the intensity of PV staining indicates the maturity level of hippocampal neurons, where immature cells have lower PV intensity than mature cells (Donato et al., 2013). We first visualized the frequency of LA/BA cells as a function of their PV staining intensity. We did not observe differences in the distributions between juvenile *Neto2*^−/−^ and *Neto2*^+/+^ mice (Fig. 2*A*, *p *=* *0.60), while the distribution of the adult *Neto2*^−/−^ mice was shifted toward low PV intensity levels compared with *Neto2*^+/+^ mice (Fig. 2*B*, *p *=* *8.94E-5). As expected, juvenile mice had lower mean PV intensity levels compared with adult mice (*Neto2*^+/+^ *p *=* *4.1E-11 and *Neto2*^−/−^ *p *=* *0.008; Fig. 2*C*). Crucially, PV intensity was significantly lower in adult, but not in juvenile, *Neto2*^−/−^ compared with *Neto2*^+/+^ mice (Fig. 2*C*, *p *=* *0.012). Because LA and BA PV interneurons may differentially influence fear learning (Lucas et al., 2016), we further investigated PV staining intensity separately in the LA and BA. BA had more PV^+^ cells (92–95% of all PV^+^ cells) than LA, and reduced PV intensity was only observed in the BA, but not in the LA of adult *Neto2*^−/−^ mice (adult *Neto2*^−/−^ vs *Neto2^+/+^* LA: *p *=* *0.99, BA: *p *=* *0.002; Fig. 2*D*,*E*). Taken together, these results indicate that NETO2 is required for proper maturation of the BA PV inhibitory network.

### Increased excitability and spine density in the *Neto2*^−/−^ BA

**Figure 3.**
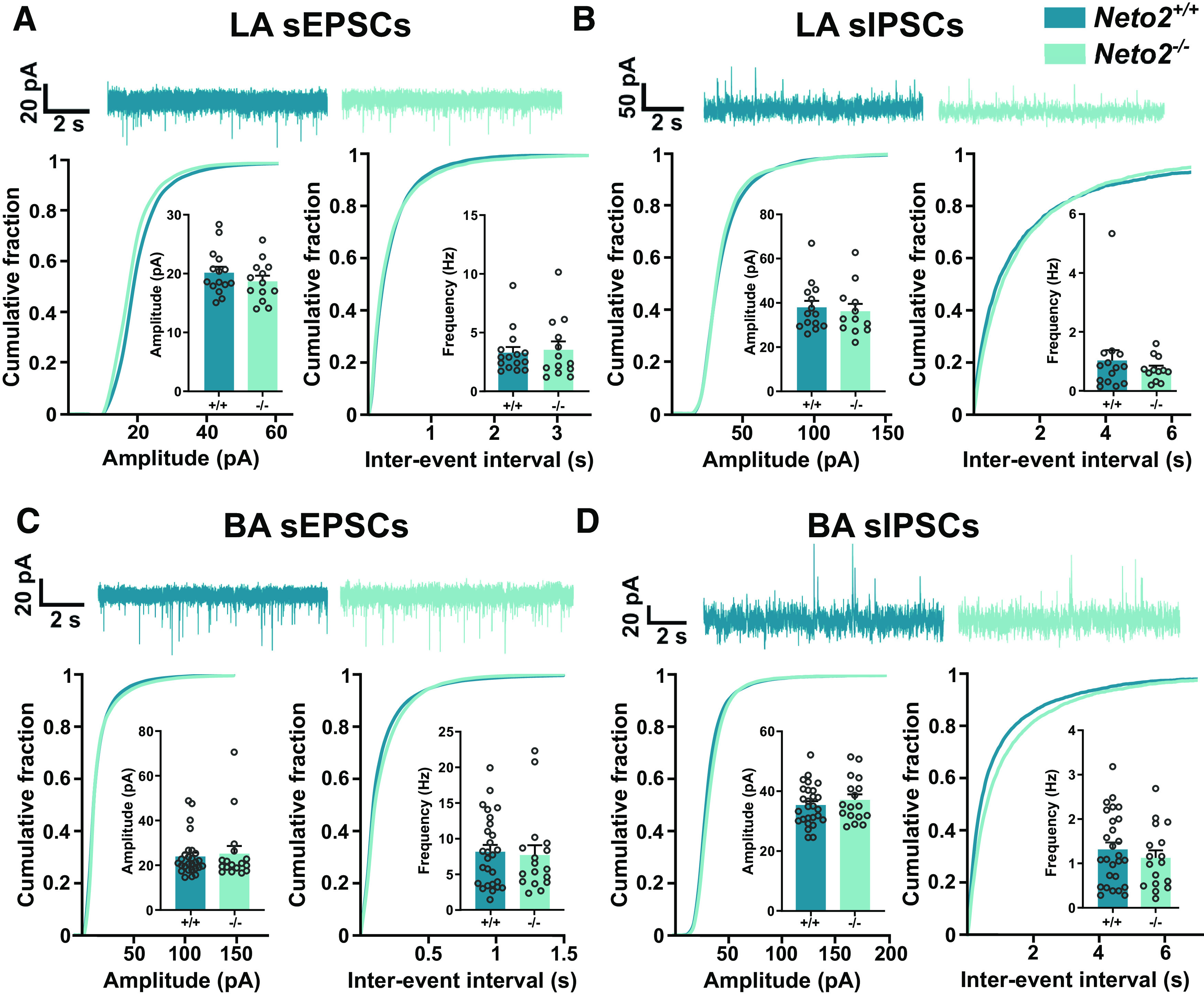
Spontaneous activity of glutamatergic and GABAergic currents in the LA and BA *Neto2*^−/−^ amygdala. Representative traces of sEPSC (***A***) and sIPSC (***B***) recordings in the LA of *Neto2*^−/−^ and *Neto2*^+/+^ mice with cumulative fraction and bar graphs for current amplitude and frequency (mice: WT *n* = 5, KO *n* = 3; recorded cells: WT *n* = 15, KO *n* = 13). Representative traces of sEPSCs (***C***) and sIPSCs (***D***) in the BA of *Neto2*^−/−^ and *Neto2*^+/+^ mice with cumulative fraction and bar graphs for current amplitude and frequency (mice: WT *n* = 6, KO *n* = 3; recorded cells: WT *n* = 27, KO *n* = 17). Each dot represents one cell. Mean ± SEM is shown. Genotype effect calculated using two-sample Kolmogorov–Smirnov test (cumulative plots) or GEE analysis (bar graphs).

To study whether the PV inhibitory network differences between adult *Neto2*^−/−^ and *Neto2*^+/+^ mice are associated with changes in the synaptic transmission in the amygdala, we recorded spontaneous glutamatergic and GABAergic synaptic currents (sEPSCs and sIPSCs) in the LA and BA (Fig. 3). *Neto2*^−/−^ and *Neto2*^+/+^ mice did not differ in either mean amplitude or frequency of sEPSCs or sIPSCs (Fig. 3), or their ratios (excitation–inhibition balance; data not shown).

**Figure 4.**
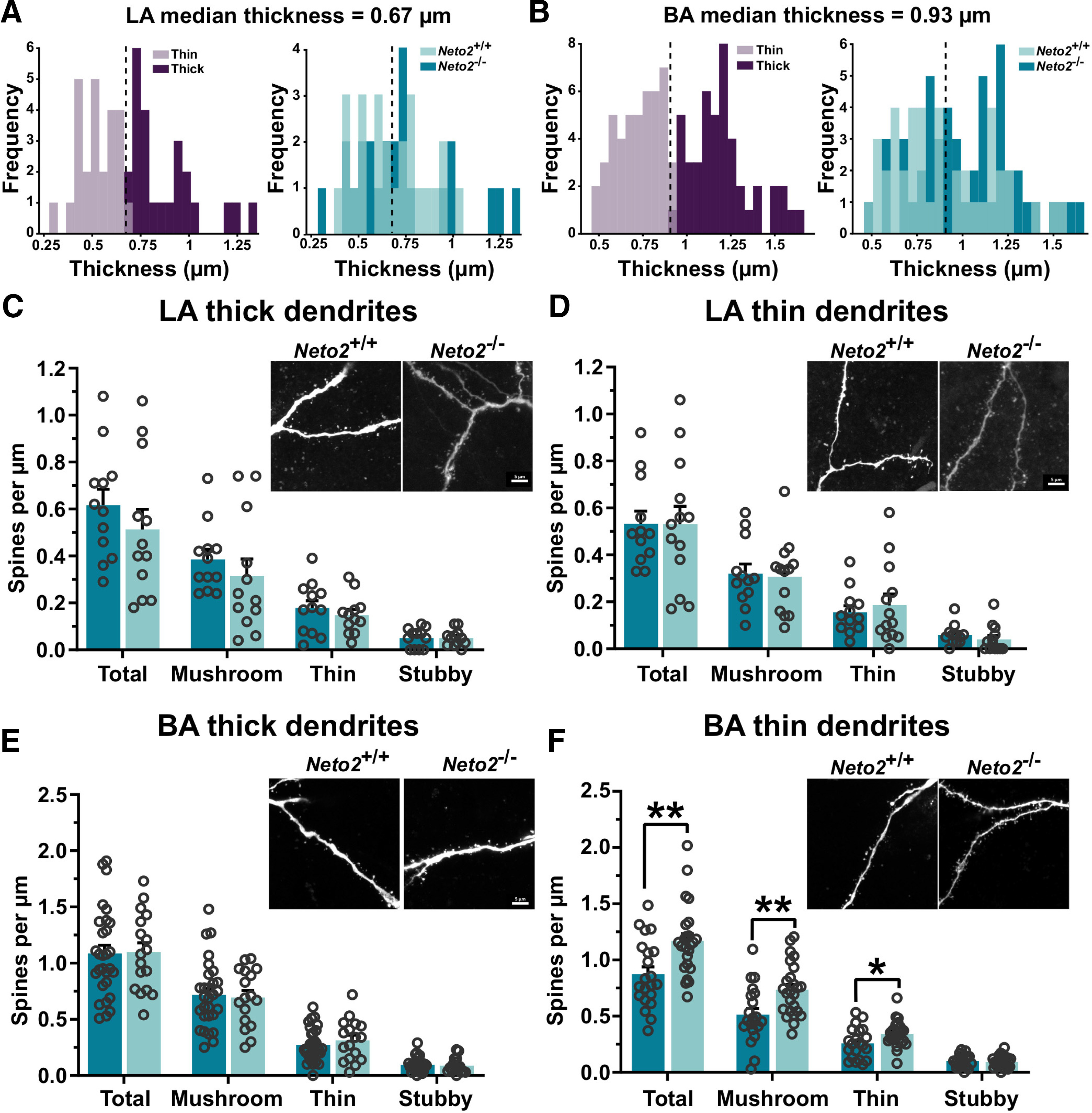
Increased dendritic spine density in the BA of *Neto2*^−/−^ mice. Frequency plot of the dendrite thickness distribution, and division into thin and thick dendrite groups in (***A***) LA and (***B***) BA of adult *Neto2*^+/+^ and *Neto2*^−/−^ mice. Density of spines within LA (***C***) thick (analyzed dendrites: WT *n* = 12, KO *n* = 12) and (***D***) thin dendrite groups (analyzed dendrites: WT *n* = 12, KO *n* = 13) from adult *Neto2*^−/−^ and *Neto2*^+/+^ mice (mice: WT *n* = 5, KO *n* = 3). Density of spines within BA (***E***) thick (analyzed dendrites: WT *n* = 29, KO *n* = 17) and (***F***) thin dendrite groups (analyzed dendrites: WT *n* = 21, KO *n* = 25) of *Neto2*^−/−^ and *Neto2*^+/+^ adult mice (mice: WT *n* = 10, KO *n* = 6). Each dot represents one dendrite. Mean ± SEM is shown. Genotype effect calculated using two-sample Kolmogorov–Smirnov test (***A***, ***B***) or GEE analysis (***C–F***); *p <* *0.05, **p <* *0.01.

To test whether dendritic spine density differs between *Neto2*^−/−^ and *Neto2*^+/+^ mice, we filled the recorded cells from LA and BA with biocytin and quantified spine numbers and densities (Fig. 4). We observed large heterogeneity in the first branch dendrite diameter, the part of the dendritic tree used for the measurements and found them to follow a bimodal distribution both in the LA and BA (Fig. 4*A*,*B*). However, these distributions did not differ between *Neto2*^−/−^ and *Neto2*^+/+^ mice (Fig. 4*A*,*B*). Because of the bimodal distribution, we examined separately the thin and thick dendrite groups divided by the median of the dendrite thickness. *Neto2*^−/−^ and *Neto2*^+/+^ mice had similar spine densities in both thin and thick dendrite groups in the LA (Fig. 4*C*,*D*). However, in the BA, spine density was higher in the thin, but not in the thick dendrite group, in *Neto2*^−/−^ compared with *Neto2*^+/+^ mice (all spine classes combined *p *=* *0.003; Fig. 4*E*,*F*). Morphologic classification of spines demonstrated that this increased density was because of a higher number of mushroom and thin spines in *Neto2*^−/−^ compared with *Neto2*^+/+^ mice (mushroom *p *=* *0.006, thin *p *=* *0.041; Fig. 4*F*).

**Figure 5.**
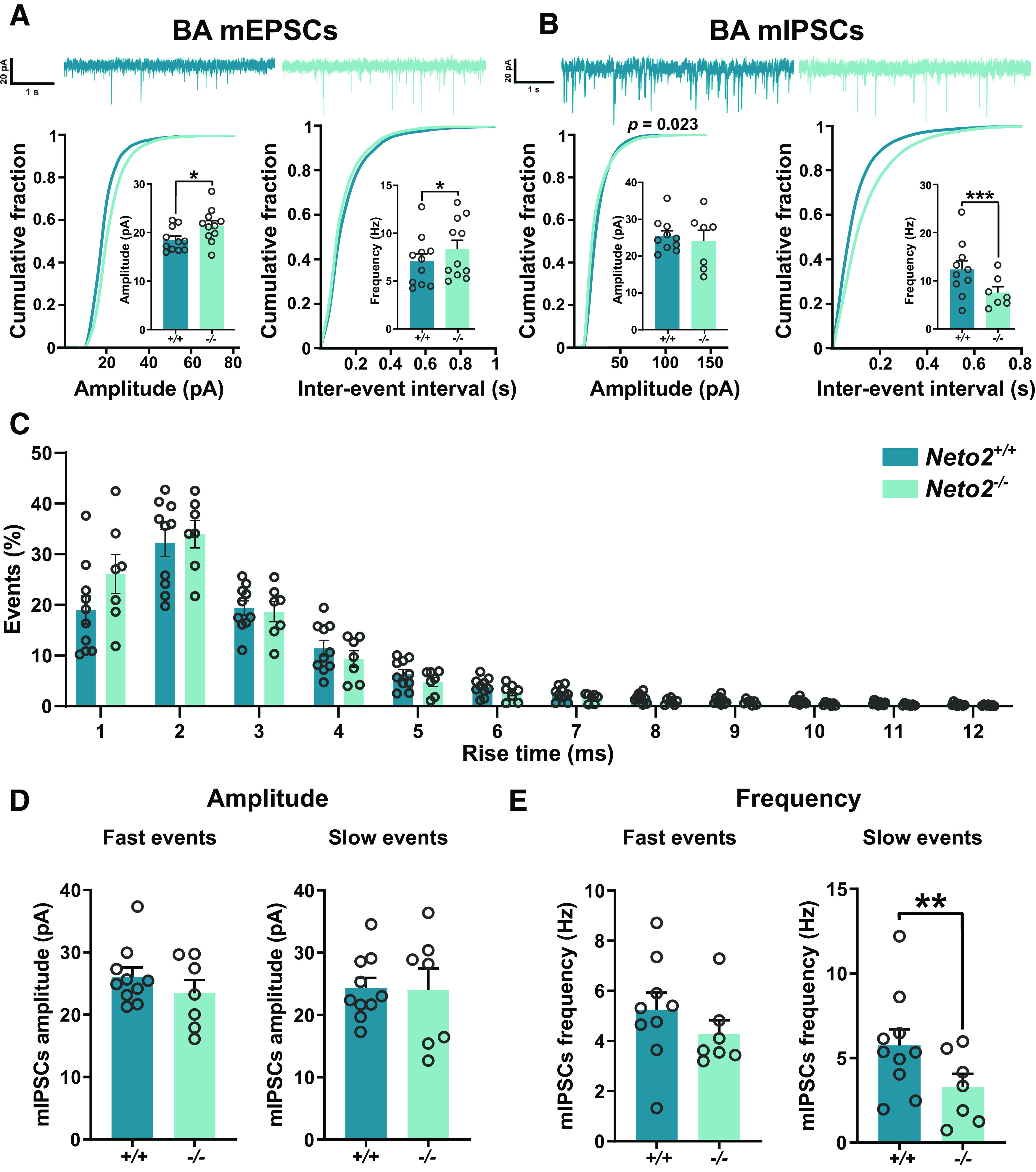
Impaired BA GABAergic transmission in *Neto2*^−/−^ mice. Representative traces of mEPSCs (***A***) and mIPSCs (***B***) with cumulative fraction and bar graphs of recorded current amplitude and frequency in the BA of adult *Neto2*^−/−^ and *Neto2*^+/+^ mice (mEPSCs: mice WT *n* = 4 and KO *n* = 3, recorded cells WT *n* = 11 and KO *n* = 11; mIPSCs: mice WT *n* = 5 and KO *n* = 3, recorded cells WT *n* = 10 and KO *n* = 7). ***C***, Histogram of mIPSCs as a function of rise-time. Fast (<2 ms) and slow (>2 ms) rise-time mIPSC event amplitude (***D***) and frequency (***E***). Each dot represents one cell. Mean ± SEM is shown. Genotype effect calculated using two-sample Kolmogorov–Smirnov test (cumulative plots and histogram) or GEE analysis (bar graphs); *p <* *0.05, **p <* *0.01, ***p <* *0.001.

Because local increased strength of glutamatergic synapses might be compensated on the network level and thus not be detectable in spontaneous activity measurements, we measured action potential-independent mEPSCs in the BA (Fig. 5*A*). Both the mean amplitude and frequency of mEPSCs were higher in *Neto2*^−/−^ compared with *Neto2*^+/+^ mice (amplitude *p *=* *0.013 and frequency *p *=* *0.039; Fig. 5*A*). Cumulative plots did not reveal significant differences in the amplitude or frequency distribution of the mEPSCs between *Neto2*^−/−^ and *Neto2*^+/+^ mice (*p *=* *0.99 and *p *=* *0.66 for amplitude and interevent interval, respectively; Fig. 5*A*). To test whether the amygdala PV interneuron immaturity observed by IHC is associated with changes in GABAergic transmission, we measured mIPSCs from BA neurons (Fig. 5*B–E*). While the mIPSC amplitude did not differ between *Neto2*^−/−^ and *Neto2*^+/+^ mice, their mean frequency was strongly decreased in the *Neto2*^−/−^ mice (Fig. 5*B*, *p *=* *6.5E-9) demonstrating either a lower release of GABA or less GABAergic innervation in BA neurons. In addition, the amplitude distribution of mIPSCs was shifted toward low amplitude events in *Neto2*^−/−^ mice (*p *=* *0.023), while their frequency distributions were similar in *Neto2*^−/−^ and *Neto2*^+/+^ mice (Fig. 5*B*). We further attempted to discriminate the origin of GABAergic inputs based on mIPSC kinetics. PV interneurons innervate the perisomatic region of amygdala principal neurons and are therefore characterized by fast rise-time (Veres et al., 2017). To elucidate the possible contribution of PV interneurons to GABAergic innervation of BA neurons, we sorted the mIPSCs in 1-ms bins based on rise-time. The relative rise-time distribution of mIPSCs did not significantly differ between *Neto2*^−/−^ and *Neto2*^+/+^ mice (Fig. 5*C*, *p *=* *0.85). We then divided the events into two groups based on their rise-time (Fig. 5*D*,*E*). The amplitudes of slow (rise-time > 2 ms) and fast rise-time mIPSCs (rise-time < 2 ms) were similar in *Neto2*^−/−^ and *Neto2*^+/+^ mice (*p *=* *0.13 and *p *=* *0.92, respectively; Fig. 5*D*). Similarly, the frequency of fast mIPSCs was equal in *Neto2*^−/−^ and *Neto2*^+/+^ mice (Fig. 5*E*, *p *=* *0.22). In contrast, the frequency of slow mIPSCs was significantly lower in *Neto2*^−/−^ compared with *Neto2*^+/+^ mice (Fig. 5*E*, *p *=* *0.006), indicating that GABAergic inputs from putative non-PV interneuron subclass is altered in *Neto2*^−/−^ mice. Altogether, these results demonstrate higher BA glutamatergic synaptic strength and lower local inhibition in *Neto2*^−/−^ mice. While our findings suggest impaired GABAergic transmission in *Neto2*^−/−^ mice, rise-time analysis of the events suggests that these changes cannot be attributed to perisomatic PV interneurons but mainly involve more distal GABAergic inputs.

### Stronger neuronal activation in the *Neto2*^−/−^ amygdala after fear acquisition

**Figure 6.**
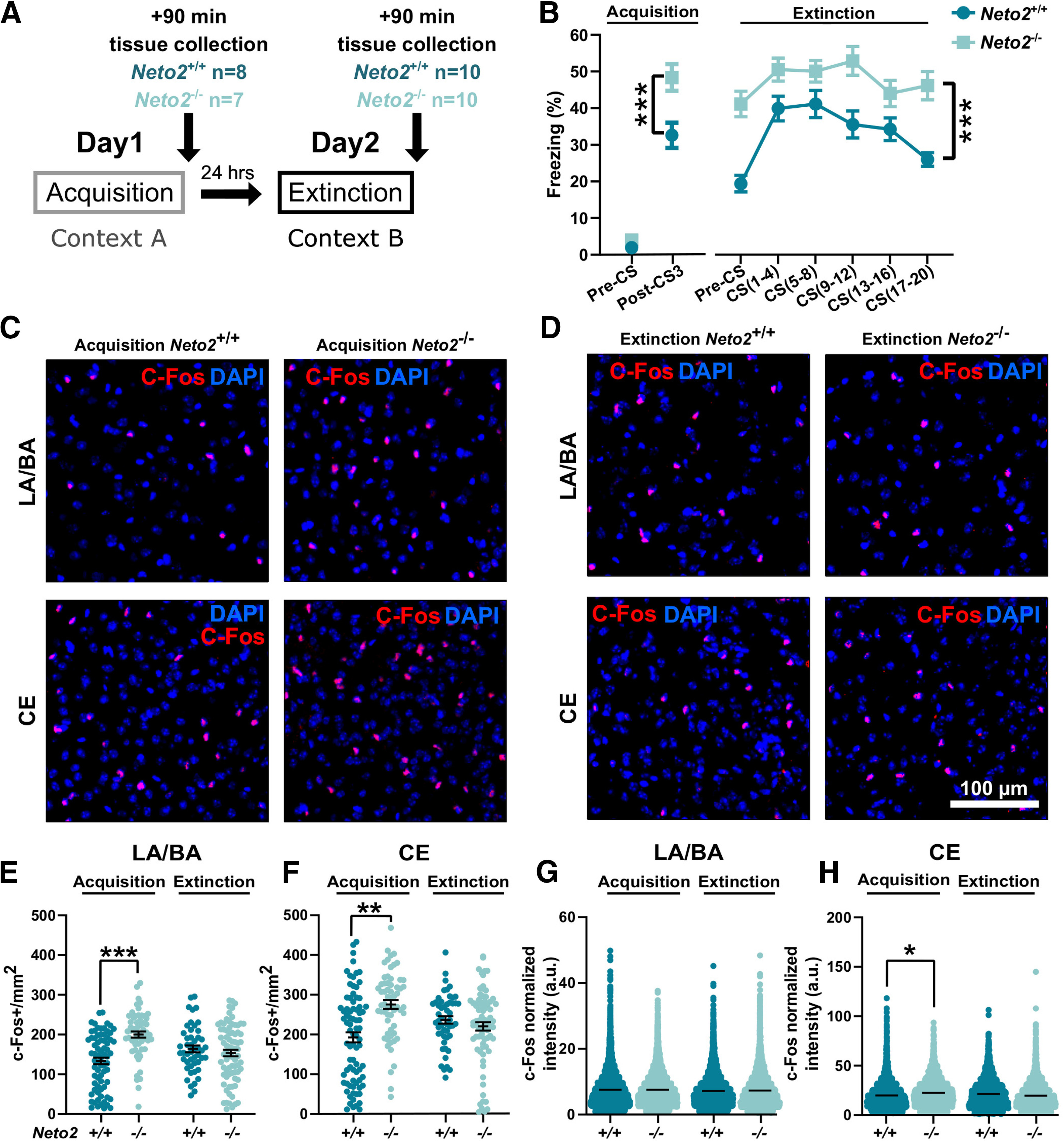
Enhanced fear-induced c-Fos activation in *Neto2*^−/−^ amygdala. ***A***, Cued fear conditioning and sample collection schedule. ***B***, Freezing percentage of adult *Neto2*^−/−^ and *Neto2*^+/+^ mice during acquisition and within-session extinction of cued fear conditioning (WT *n* = 42, KO *n* = 39). *Neto2*^−/−^ mice froze significantly more than *Neto2*^+/+^ mice already after the third US–CS pairing (Post-CS3), and they were unable to extinguish the fear memory measured the following day after 20 CS presentations. We collected brain tissue from a subset of these mice 90 min after the end of fear acquisition or extinction and performed c-Fos staining (acquisition: WT *n* = 9, KO *n* = 8 and extinction: WT *n* = 10, KO *n* = 10). Close-up representative images of c-Fos staining in the amygdala of *Neto2*^+/+^ and *Neto2*^−/−^ mice after (***C***) fear acquisition or (***D***) extinction. Quantification of c-Fos^+^ cells per mm^2^ (c-Fos^+^/mm^2^) in (***E***) LA/BA (analyzed images: acquisition WT *n* = 73, KO *n* = 59; extinction WT *n* = 48, KO *n* = 78) and (***F***) CE (analyzed images: acquisition WT *n* = 80, KO *n* = 61; extinction WT *n* = 51, KO *n* = 78). Mean intensity of c-Fos^+^ cells in (***G***) LA/BA (analyzed cells: acquisition WT *n* = 5453, KO *n* = 7146; extinction WT *n* = 5052, KO *n* = 6954) and (***H***) CE (analyzed cells: acquisition WT *n* = 2928, KO *n* = 3317; extinction WT *n* = 2461, KO *n* = 3524). Each dot represents data from one image (***E***, ***F***) or one cell (***G***, ***H***). Mean ± SEM is shown. Genotype effect calculated using mixed ANOVA (behavior) and GEE analysis (c-Fos); *p <* *0.05, **p <* *0.01, ***p <* *0.001.

The amygdala subnuclei function differentially in fear learning and memory. LA and BA receive fear stimuli and store fear memories, respectively, while CE is the output nucleus for defensive behaviors, such as freezing. To investigate neuronal activation of the amygdala after fear acquisition or extinction, we quantified the number of cells expressing the c-Fos immediate early gene in the LA/BA or in the CE in *Neto2*^−/−^ and *Neto2*^+/+^ mice (Fig. 6). At the behavioral level, we replicated the earlier findings (Mennesson et al., 2019) demonstrating that *Neto2*^−/−^ mice have higher fear expression and impaired extinction compared with *Neto2*^+/+^ mice during cued fear conditioning [acquisition *Neto2*^−/−^ vs *Neto2*^+/+^ pre-CS: *p *=* *0.59 and post-CS3: *p *=* *4.54E-05; extinction time (CS) effect: *p *=* *1.28E-10, genotype effect: *p *=* *3.71E-05, time (CS) × genotype interaction effect: *p *=* *0.00011. CS(1–4) vs CS(17–20): *Neto2*^+/+^ *p *=* *8.13E-05 and *Neto2*^−/−^ *p *=* *0.22; Fig. 6*B*]. *Neto2*^−/−^ mice had a larger number of c-Fos^+^ cells in both LA/BA (*p *=* *0.00009) and CE (*p *= 0.0048) after fear acquisition, but not after fear extinction, compared with *Neto2*^+/+^ mice (Fig. 6*C–F*).

In the hippocampus, the intensity of c-Fos staining predicts the strength of the acquired fear learning after contextual fear conditioning (Ruediger et al., 2011), but it is not known whether similar mechanisms apply to cued fear conditioning in the amygdala. To test whether *Neto2*^−/−^ mice, have higher fear expression than *Neto2*^+/+^ mice, also have higher c-Fos intensity, we measured the average intensity of c-Fos after fear acquisition and extinction. The intensity of c-Fos staining in the CE was higher in the *Neto2*^−/−^ mice after fear acquisition (*p *=* *0.035) compared with the *Neto2*^+/+^ mice, while there were no differences between genotypes in the LA/BA or after extinction (Fig. 6*G*,*H*). Taken together, these results indicate stronger neuronal activation of LA, BA, and CE immediately after the acquisition of CS–US association in *Neto2*^−/−^ mice and possibly an increased strength of fear memory as shown by the higher intensity of c-Fos staining in the CE. Since the CE is critical for the execution of defensive behaviors, its stronger activation may underlie the higher freezing levels observed in mice lacking NETO2.

## Discussion

In this study, we examined PV interneuron network maturity of the amygdala as a potential mediator of higher fear expression and impaired extinction observed in *Neto2*^−/−^ mice. We found that adult, but not juvenile, *Neto2*^−/−^ mice had less PV^+^PNN^+^ cells relative to the total population of PNN^+^ cells and lower PV staining intensity, suggesting immaturity and increased plasticity of the *Neto2*^−/−^ amygdala. These findings were associated with a decrease in mIPSC frequency in BA neurons. In addition, we found an increase in mEPSC amplitude and frequency in the BA cells and higher spine density of thin BA dendrites in *Neto2*^−/−^ adult mice, indicating stronger glutamatergic synapses in the absence of NETO2. We also found a larger number of c-Fos^+^ cells in *Neto2*^−/−^ mice undergoing associative fear learning, suggesting stronger neuronal recruitment associated with fear acquisition.

We observed that the fraction of the PV^+^PNN^+^ double positive cells relative to the total population of PNN^+^ cells was significantly lower in adult, but not juvenile, *Neto2*^−/−^ compared with *Neto2*^+/+^ mice. Chronic fluoxetine treatment, which re-opens critical period-like plasticity in adult mouse brain, causes similar reduction in the fraction of the PV^+^PNN^+^ cells relative to the total population of PNN^+^ cells (Karpova et al., 2011). Thus, our finding indicates that the PV interneurons in the adult *Neto2*^−/−^ amygdala remain in an immature state. We did not detect genotype differences in the numbers of PV^+^ or PNN^+^ single positive cells. The reliability of immunohistochemical analyses relies on the quality of the used antibodies and how quantitatively they are able to detect the correct antigen. For this reason, we may have missed cells that express very low levels of PV. Despite these challenges, also the PV staining intensity has been used as a molecular plasticity marker, with low PV staining intensity correlating with high levels of plasticity, and the staining intensity increasing with age (Donato et al., 2013, 2015; Umemori et al., 2018). PV intensity levels have also been associated with significant behavioral changes, as shown for environmental enrichment (shift toward low PV intensity) and fear conditioning (shift toward high PV intensity) in the mouse hippocampus (Donato et al., 2013). Inspired by these earlier studies using the same antibody as we used, we measured PV staining intensity in the amygdala. As expected, PV intensity was lower in juvenile than adult WT mice. At P23, PV intensities were similar in *Neto2*^−/−^ and *Neto2*^+/+^ mice. However, adult *Neto2*^−/−^ mice had significantly lower PV intensity than *Neto2*^+/+^ mice, indicating that the maturation of the amygdala PV inhibitory network is perturbed between juvenile and adult ages in the absence of NETO2.

In support of perturbed GABAergic inhibition, mIPSC frequency in BA neurons was significantly lower in *Neto2*^−/−^ as compared with *Neto2*^+/+^ mice. However, this change was mainly because of loss of GABAergic events with a slow rise-time, that are expected to originate from synapses innervating distal dendrites. Since PV interneurons predominantly innervate perisomatic region of the principal neurons (Veres et al., 2017), this result suggests that the absence of NETO2 influences not only PV interneurons but also other subtypes of GABAergic neurons in the amygdala. In the hippocampus, presynaptic KARs regulate GABA release from cholecystokinin (CCK)-positive interneurons depending on the presence of NETO2 (Wyeth et al., 2017), and a comparable mechanism could contribute to the observed decrease in GABAergic transmission in the BA of *Neto2*^−/−^ mice. The lack of apparent changes in the frequency of fast, expectedly PV neuron dependent mIPSCs is not necessarily in contrast with the histologic results indicating low intensity of PV immunostaining in the amygdala of *Neto2*^−/−^ mice. PV is a presynaptic calcium binding protein that regulates neurotransmitter release, and in the absence of endogenous PV, asynchronous GABA release is enhanced (Manseau et al., 2010; Veres et al., 2017). Therefore, increased release because of low PV content might compensate for any effect of reduced GABAergic innervation from PV interneurons, resulting in overall change in the mean mIPSC frequency. In the amygdala, PV interneurons gate plasticity of LA/BA principal neurons and they are crucial for fear memory acquisition during associative fear learning (Wolff et al., 2014). Therefore, an immature and more plastic PV inhibitory network in the amygdala, like in the absence of NETO2, could lead to imbalanced inhibition of amygdala principal neurons during fear learning and consequently result in higher fear expression.

In addition, we found stronger glutamatergic synapses in the BA in the absence of NETO2. Adult *Neto2*^−/−^ mice had higher amplitude and frequency of mEPSCs in the BA compared with *Neto2*^+/+^ mice. In line with this finding, spine density of thin, but not the thick, dendrites was higher in the BA of *Neto2*^−/−^ compared with *Neto2*^+/+^ mice. Morphologically, neurons from the amygdala are variable in shape and have numerous expansions and projections (McDonald, 1982; Pitkänen et al., 2003), but it is not known whether the diameter of the dendrite is related to their maturity. Whether the cells with thick and thin dendrites represent different classes of neurons with different functions remain to be determined. The increase in the strength of glutamatergic connectivity did not significantly affect the basal excitation–inhibition balance (i.e., sEPSC and sIPSC frequency) of the BA network *in vitro*, possibly because chronic changes in network excitability are efficiently compensated for by various homeostatic mechanisms (Davis, 2013). However, these results do not exclude the possibility that NETO2 deficiency affects recruitment of interneurons during network activity in vivo, which would affect excitation–inhibition balance in the amygdala in response to sensory activity and during behavioral tasks. Consistent with this idea, we did find significantly higher abundance of c-Fos^+^ cells, a widely used marker for neuronal activation (Chung, 2015), in both LA/BA and CE after fear acquisition in *Neto2*^−/−^ compared with *Neto2*^+/+^ mice, indicative of higher excitability of the amygdala during a behavioral task. In the BA, the number of c-Fos^+^ cells positively correlates with fear expression of conditioned fear during memory retrieval (Reijmers et al., 2007). It is therefore possible that the increased number of c-Fos^+^ cells in the *Neto2*^−/−^ mice after fear acquisition may be related to stronger associative learning, which is consistent with their higher fear expression phenotype. Thus, NETO2 may facilitate fear acquisition and thereby influence expression of conditioned fear.

Although fear extinction was delayed in the *Neto2*^−/−^ mice, we observed no differences in the number of c-Fos^+^ cells between genotypes after fear extinction. Fear acquisition and extinction depend on different neuronal populations within the mPFC-amygdala-vHpc network (Herry et al., 2008; Senn et al., 2014). BA principal neurons are central for fear learning and extinction. During fear learning, fear neurons of the BA receive input from the vHpc and project to the mPFC, while reciprocal connection between BA extinction neurons and mPFC are necessary for fear extinction (Herry et al., 2008). Especially the IL subregion of the mPFC is required for fear extinction (Cho et al., 2013; Bloodgood et al., 2018). Because of the differential recruitment of these connections during fear learning and extinction, the delayed fear extinction of *Neto2*^−/−^ mice may originate from deficient neuronal recruitment and/or activity of the mPFC neuronal population that projects to the amygdala rather than from a defect in the amygdala microcircuitry. This is in line with neuroimaging studies of PTSD patients, in which loss of inhibitory control resulting from mPFC hypoactivation, has been suggested to cause hyperactivation of the amygdala and hippocampal atrophy (Shin et al., 2006; Kamiya and Abe, 2020).

Our study has several limitations, and it left open questions to be addressed in future work. Although our results of the immaturity and hyperactivity of the amygdala in the absence of NETO2 are consistent with the higher fear expression and extinction deficit of *Neto2*^−/−^ mice, we have not been able to establish causal links between the main individual findings (i.e., the immature PV network, stronger glutamatergic synapses associated with increased spine density, and increased neuronal activation on fear acquisition) in this article. *Neto2* is widely expressed in the fear-associated brain regions, both in the excitatory and inhibitory neurons (Mennesson et al., 2019). Within the developing hippocampus, *Neto2* is expressed in PV^+^, SOM^+^, and CCK/cannabinoid receptor 1 (CCK/CB1^+^)-expressing interneurons together with GluK1, GluK2, and GluK5 subunits of KAR (Wyeth et al., 2017). This wide expression pattern makes it challenging to establish causal associations. Very little is known about cell-type-specific functions of NETO2. In the hippocampus, it promotes tonic presynaptic KAR activation and increases inhibition mediated by the CCK/CB1^+^ neurons (Wyeth et al., 2017). In addition to KARs, NETO2 interacts with KCC2 and together with GluK2 increases the total KCC2 abundance (Pressey et al., 2017). Given the wide expression pattern and multiple possible interaction partners, cell-type specific molecular and electrophysiological approaches would be helpful in determining the mechanisms underlying the fear phenotypes of the *Neto2*^−/−^ mice. We used a total KO model of *Neto2*, which did not allow us to investigate, for example, whether the morphologic changes of the dendritic spines derive from presynaptic and/or postsynaptic actions of NETO2. Finally, our data suggest that in addition to the amygdala PV network, NETO2 influences also other GABAergic neurons, and these mechanisms remain to be determined.

In conclusion, we established that the higher fear expression and delayed extinction phenotype observed in adult *Neto2*^−/−^ mice is associated with immature features of PV interneurons and stronger glutamatergic synapses within the amygdala. Moreover, neuronal activation of the amygdala was increased after fear learning in the absence of NETO2, possibly leading to stronger associative memories, consistent with higher fear expression after acquisition. However, the higher amygdala excitability derives from other than PV inputs to the pyramidal cells, and the mechanistic basis of this finding remains to be studied in the future. Higher fear expression has been associated with higher conditionability, which together with slower extinction, is a phenotype observed in human PTSD (Orr et al., 2000; Blechert et al., 2007; Wegerer et al., 2013). Therefore, *Neto2*^−/−^ mice may be used as a model for PTSD risk. This model could be especially useful for studying the role of maturity and plasticity of the amygdala in fear-related disorders.

## Synthesis

Reviewing Editor: Carmen Sandi, Swiss Federal Institute of Technology Decisions are customarily a result of the Reviewing Editor and the peer reviewers coming together and discussing their recommendations until a consensus is reached. When revisions are invited, a fact-based synthesis statement explaining their decision and outlining what is needed to prepare a revision will be listed below. The following reviewer(s) agreed to reveal their identity: Carsten Wotjak.

This study provides novel insight into the mechanisms of excitation/inhibition balance in the amygdala related to fear behavior by highlighting the role of NETO2 in proper amygdala interneuron development. The authors have addressed our previous concerns to our satisfaction. In this revised ms., the authors have expanded the electrophysiological analyses by including data demonstrating decreased inhibitory input onto BLA pyramidal cells. These data provide a more complete picture of the circuit alterations in the Neto2-/- mice. The new results are well presented and fairly discussed, including the possibility that deficient GABAergic input may originate from non-PV interneurons. The new data on mIPSCs significantly strengthens the conclusion of the study. Our only remaining (minor) concern relates to the data shown in Fig. 1e: As indicated by the alternative Fig. 1e, these data do not seem to be normally distributed (due to a ceiling effect). We would like to ask the authors to redo the test of normality and to readjust the statistical analysis, if necessary.

In addition, we recommend to use the alternative Fig. 1. For the other figures, we favor the option with single datapoints or violin plots offered in the alternative version submitted with the revision (optional).

## References

[B1] Beurdeley M, Spatazza J, Lee HH, Sugiyama S, Bernard C, Di Nardo AA, Hensch TK, Prochiantz A (2012) Otx2 binding to perineuronal nets persistently regulates plasticity in the mature visual cortex. J Neurosci 32:9429–9437. 10.1523/JNEUROSCI.0394-12.2012 22764251 PMC3419577

[B2] Blechert J, Michael T, Vriends N, Margraf J, Wilhelm FH (2007) Fear conditioning in posttraumatic stress disorder: evidence for delayed extinction of autonomic, experiential, and behavioural responses. Behav Res Ther 45:2019–2033. 10.1016/j.brat.2007.02.012 17442266

[B3] Bloodgood DW, Sugam JA, Holmes A, Kash TL (2018) Fear extinction requires infralimbic cortex projections to the basolateral amygdala. Transl Psychiatry 8:60. 10.1038/s41398-018-0106-x 29507292 PMC5838104

[B4] Carstens KE, Phillips ML, Pozzo-Miller L, Weinberg RJ, Dudek SM (2016) Perineuronal nets suppress plasticity of excitatory synapses on CA2 pyramidal neurons. J Neurosci 36:6312–6320. 10.1523/JNEUROSCI.0245-16.2016 27277807 PMC4899529

[B5] Cho JH, Bayazitov IT, Meloni EG, Myers KM, Carlezon WA Jr, Zakharenko SS, Bolshakov VY (2011) Coactivation of thalamic and cortical pathways induces input timing-dependent plasticity in amygdala. Nat Neurosci 15:113–122. 10.1038/nn.2993 22158512 PMC3245819

[B6] Cho JH, Deisseroth K, Bolshakov VY (2013) Synaptic encoding of fear extinction in mPFC-amygdala circuits. Neuron 80:1491–1507. 10.1016/j.neuron.2013.09.025 24290204 PMC3872173

[B7] Chung L (2015) A brief introduction to the transduction of neural activity into Fos signal. Dev Reprod 19:61–67. 10.12717/DR.2015.19.2.061 27004262 PMC4801051

[B8] Davis GW (2013) Homeostatic signaling and the stabilization of neural function. Neuron 80:718–728. 10.1016/j.neuron.2013.09.044 24183022 PMC3856728

[B9] Donato F, Rompani SB, Caroni P (2013) Parvalbumin-expressing basket-cell network plasticity induced by experience regulates adult learning. Nature 504:272–276. 10.1038/nature12866 24336286

[B10] Donato F, Chowdhury A, Lahr M, Caroni P (2015) Early- and late-born parvalbumin basket cell subpopulations exhibiting distinct regulation and roles in learning. Neuron 85:770–786. 10.1016/j.neuron.2015.01.011 25695271

[B11] Ehrlich I, Humeau Y, Grenier F, Ciocchi S, Herry C, Lüthi A (2009) Amygdala inhibitory circuits and the control of fear memory. Neuron 62:757–771. 10.1016/j.neuron.2009.05.026 19555645

[B12] Fitzgerald PJ, Pinard CR, Camp MC, Feyder M, Sah A, Bergstrom HC, Graybeal C, Liu Y, Schlüter OM, Grant SG, Singewald N, Xu W, Holmes A (2015) Durable fear memories require PSD-95. Mol Psychiatry 20:901–912. 10.1038/mp.2014.161 25510511 PMC4469631

[B13] Gogolla N, Caroni P, Lüthi A, Herry C (2009) Perineuronal nets protect fear memories from erasure. Science 325:1258–1261. 10.1126/science.1174146 19729657

[B14] Guirado R, Perez-Rando M, Sanchez-Matarredona D, Castrén E, Nacher J (2014) Chronic fluoxetine treatment alters the structure, connectivity and plasticity of cortical interneurons. Int J Neuropsychopharmacol 17:1635–1646. 10.1017/S1461145714000406 24786752

[B15] Gunduz-Cinar O, Brockway E, Lederle L, Wilcox T, Halladay LR, Ding Y, Oh H, Busch EF, Kaugars K, Flynn S, Limoges A, Bukalo O, MacPherson KP, Masneuf S, Pinard C, Sibille E, Chesler EJ, Holmes A (2019) Identification of a novel gene regulating amygdala-mediated fear extinction. Mol Psychiatry 24:601–612. 10.1038/s41380-017-0003-3 29311651 PMC6035889

[B16] Hanley JA, Negassa A, Edwardes MD, Forrester JE (2003) Statistical analysis of correlated data using generalized estimating equations: an orientation. Am J Epidemiol 157:364–375. 10.1093/aje/kwf215 12578807

[B17] Herry C, Johansen JP (2014) Encoding of fear learning and memory in distributed neuronal circuits. Nat Neurosci 17:1644–1654. 10.1038/nn.3869 25413091

[B18] Herry C, Ciocchi S, Senn V, Demmou L, Müller C, Lüthi A (2008) Switching on and off fear by distinct neuronal circuits. Nature 454:600–606. 10.1038/nature07166 18615015

[B19] Herry C, Ferraguti F, Singewald N, Letzkus JJ, Ehrlich I, Lüthi A (2010) Neuronal circuits of fear extinction. Eur J Neurosci 31:599–612. 10.1111/j.1460-9568.2010.07101.x 20384807

[B20] Ivakine EA, Acton BA, Mahadevan V, Ormond J, Tang M, Pressey JC, Huang MY, Ng D, Delpire E, Salter MW, Woodin MA, McInnes RR (2013) Neto2 is a KCC2 interacting protein required for neuronal Cl- regulation in hippocampal neurons. Proc Natl Acad Sci USA 110:3561–3566. 10.1073/pnas.1212907110 23401525 PMC3587235

[B21] Johansen JP, Hamanaka H, Monfils MH, Behnia R, Deisseroth K, Blair HT, LeDoux JE (2010) Optical activation of lateral amygdala pyramidal cells instructs associative fear learning. Proc Natl Acad Sci USA 107:12692–12697. 10.1073/pnas.1002418107 20615999 PMC2906568

[B22] Johansen JP, Cain CK, Ostroff LE, LeDoux JE (2011) Molecular mechanisms of fear learning and memory. Cell 147:509–524. 10.1016/j.cell.2011.10.009 22036561 PMC3215943

[B23] Kamiya K, Abe O (2020) Imaging of posttraumatic stress disorder. Neuroimaging Clin N Am 30:115–123. 10.1016/j.nic.2019.09.010 31759567

[B24] Karpova NN, Pickenhagen A, Lindholm J, Tiraboschi E, Kulesskaya N, Agústsdóttir A, Antila H, Popova D, Akamine Y, Bahi A, Sullivan R, Hen R, Drew LJ, Castrén E (2011) Fear erasure in mice requires synergy between antidepressant drugs and extinction training. Science 334:1731–1734. 10.1126/science.1214592 22194582 PMC3929964

[B25] Ko S, Zhao MG, Toyoda H, Qiu CS, Zhuo M (2005) Altered behavioral responses to noxious stimuli and fear in glutamate receptor 5 (GluR5)- or GluR6-deficient mice. J Neurosci 25:977–984. 10.1523/JNEUROSCI.4059-04.2005 15673679 PMC6725621

[B26] Laine MA, Trontti K, Misiewicz Z, Sokolowska E, Kulesskaya N, Heikkinen A, Saarnio S, Balcells I, Ameslon P, Greco D, Mattila P, Ellonen P, Paulin L, Auvinen P, Jokitalo E, Hovatta I (2018) Genetic control of myelin plasticity after chronic psychosocial stress. eNeuro 5:ENEURO.0166-18.2018 10.1523/ENEURO.0166-18.2018 PMC6071195 30073192

[B27] Li H, Chen A, Xing G, Wei ML, Rogawski MA (2001) Kainate receptor-mediated heterosynaptic facilitation in the amygdala. Nat Neurosci 4:612–620. 10.1038/88432 11369942

[B28] Lorenzo Bozzelli P, Alaiyed S, Kim E, Villapol S, Conant K (2018) Proteolytic remodeling of perineuronal nets: effects on synaptic plasticity and neuronal population dynamics. Neural Plast 2018:5735789. 10.1155/2018/5735789 29531525 PMC5817213

[B29] Lucas EK, Jegarl AM, Morishita H, Clem RL (2016) Multimodal and site-specific plasticity of amygdala parvalbumin interneurons after fear learning. Neuron 91:629–643. 10.1016/j.neuron.2016.06.032 27427462 PMC4975985

[B30] McDonald AJ (1982) Neurons of the lateral and basolateral amygdaloid nuclei: a Golgi study in the rat. J Comp Neurol 212:293–312. 10.1002/cne.902120307 6185547

[B31] Mennesson M, Rydgren E, Lipina T, Sokolowska E, Kulesskaya N, Morello F, Ivakine E, Voikar V, Risbrough V, Partanen J, Hovatta I (2019) Kainate receptor auxiliary subunit NETO2 is required for normal fear expression and extinction. Neuropsychopharmacol 44:1855–1866. 10.1038/s41386-019-0344-5 PMC6784901 30770891

[B32] Morikawa S, Ikegaya Y, Narita M, Tamura H (2017) Activation of perineuronal net-expressing excitatory neurons during associative memory encoding and retrieval. Sci Rep 7:46024. 10.1038/srep46024 28378772 PMC5380958

[B33] Nowicka D, Soulsby S, Skangiel-Kramska J, Glazewski S (2009) Parvalbumin-containing neurons, perineuronal nets and experience-dependent plasticity in murine barrel cortex. Eur J Neurosci 30:2053–2063. 10.1111/j.1460-9568.2009.06996.x 20128844

[B34] Ohira K, Takeuchi R, Iwanaga T, Miyakawa T (2013) Chronic fluoxetine treatment reduces parvalbumin expression and perineuronal nets in gamma-aminobutyric acidergic interneurons of the frontal cortex in adult mice. Mol Brain 6:43. 10.1186/1756-6606-6-43 24228616 PMC4225860

[B35] Orav E, Atanasova T, Shintyapina A, Kesaf S, Kokko M, Partanen J, Taira T, Lauri SE (2017) NETO1 guides development of glutamatergic connectivity in the hippocampus by regulating axonal kainate receptors. eNeuro 4:ENEURO.0048-17.2017 10.1523/ENEURO.0048-17.2017 PMC5494894 28680963

[B36] Orr SP, Metzger LJ, Lasko NB, Macklin ML, Peri T, Pitman RK (2000) De novo conditioning in trauma-exposed individuals with and without posttraumatic stress disorder. J Abnorm Psychol 109:290–298. 10895567

[B37] Pattwell SS, Bath KG, Casey BJ, Ninan I, Lee FS (2011) Selective early-acquired fear memories undergo temporary suppression during adolescence. Proc Natl Acad Sci USA 108:1182–1187. 10.1073/pnas.1012975108 21220344 PMC3024661

[B38] Pattwell SS, Duhoux S, Hartley CA, Johnson DC, Jing D, Elliott MD, Ruberry EJ, Powers A, Mehta N, Yang RR, Soliman F, Glatt CE, Casey BJ, Ninan I, Lee FS (2012) Altered fear learning across development in both mouse and human. Proc Natl Acad Sci USA 109:16318–16323. 10.1073/pnas.1206834109 22988092 PMC3479553

[B39] Pitkänen A, Savander M, Nurminen N, Ylinen A (2003) Intrinsic synaptic circuitry of the amygdala. Ann NY Acad Sci 985:34–49. 10.1111/j.1749-6632.2003.tb07069.x 12724146

[B40] Pressey JC, Mahadevan V, Khademullah CS, Dargaei Z, Chevrier J, Ye W, Huang M, Chauhan AK, Meas SJ, Uvarov P, Airaksinen MS, Woodin MA (2017) A kainate receptor subunit promotes the recycling of the neuron-specific K(+)-Cl(-) co-transporter KCC2 in hippocampal neurons. J Biol Chem 292:6190–6201. 10.1074/jbc.M116.767236 28235805 PMC5391750

[B41] Reijmers LG, Perkins BL, Matsuo N, Mayford M (2007) Localization of a stable neural correlate of associative memory. Science 317:1230–1233. 10.1126/science.1143839 17761885

[B42] Ruediger S, Vittori C, Bednarek E, Genoud C, Strata P, Sacchetti B, Caroni P (2011) Learning-related feedforward inhibitory connectivity growth required for memory precision. Nature 473:514–518. 10.1038/nature09946 21532590

[B43] Senn V, Wolff SB, Herry C, Grenier F, Ehrlich I, Gründemann J, Fadok JP, Müller C, Letzkus JJ, Lüthi A (2014) Long-range connectivity defines behavioral specificity of amygdala neurons. Neuron 81:428–437. 10.1016/j.neuron.2013.11.006 24462103

[B44] Shin LM, Rauch SL, Pitman RK (2006) Amygdala, medial prefrontal cortex, and hippocampal function in PTSD. Ann NY Acad Sci 1071:67–79. 10.1196/annals.1364.007 16891563

[B45] Shin RM, Tully K, Li Y, Cho JH, Higuchi M, Suhara T, Bolshakov VY (2010) Hierarchical order of coexisting pre- and postsynaptic forms of long-term potentiation at synapses in amygdala. Proc Natl Acad Sci USA 107:19073–19078. 10.1073/pnas.1009803107 20956319 PMC2973868

[B46] Tang M, Pelkey KA, Ng D, Ivakine E, McBain CJ, Salter MW, McInnes RR (2011) Neto1 is an auxiliary subunit of native synaptic kainate receptors. J Neurosci 31:10009–10018. 10.1523/JNEUROSCI.6617-10.2011 21734292 PMC3148853

[B47] Tovote P, Fadok JP, Lüthi A (2015) Neuronal circuits for fear and anxiety. Nat Rev Neurosci 16:317–331. 10.1038/nrn3945 25991441

[B48] Umemori J, Winkel F, Castrén E, Karpova NN (2015) Distinct effects of perinatal exposure to fluoxetine or methylmercury on parvalbumin and perineuronal nets, the markers of critical periods in brain development. Int J Dev Neurosci 44:55–64. 10.1016/j.ijdevneu.2015.05.006 25997908

[B49] Umemori J, Winkel F, Didio G, Llach Pou M, Castrén E (2018) iPlasticity: induced juvenile-like plasticity in the adult brain as a mechanism of antidepressants. Psychiatry Clin Neurosci 72:633–653. 10.1111/pcn.12683 29802758 PMC6174980

[B50] Manseau F, Marinelli S, Méndez P, Schwaller B, Prince DA, Huguenard JR, Bacci A (2010) Desynchronization of neocortical networks by asynchronous release of GABA at autaptic and synaptic contacts from fast-spiking interneurons. PLoS Biol 8:e1000492 10.1371/journal.pbio.1000492 20927409 PMC2946936

[B51] Veres JM, Nagy GA, Hájos N (2017) Perisomatic GABAergic synapses of basket cells effectively control principal neuron activity in amygdala networks. Elife 6:e20721 10.7554/eLife.20721 28060701 PMC5218536

[B52] Wang D, Fawcett J (2012) The perineuronal net and the control of CNS plasticity. Cell Tissue Res 349:147–160. 10.1007/s00441-012-1375-y 22437874

[B53] Wegerer M, Blechert J, Kerschbaum H, Wilhelm FH (2013) Relationship between fear conditionability and aversive memories: evidence from a novel conditioned-intrusion paradigm. PLoS One 8:e79025. 10.1371/journal.pone.0079025 24244407 PMC3828300

[B54] Wolff SB, Gründemann J, Tovote P, Krabbe S, Jacobson GA, Müller C, Herry C, Ehrlich I, Friedrich RW, Letzkus JJ, Lüthi A (2014) Amygdala interneuron subtypes control fear learning through disinhibition. Nature 509:453–458. 10.1038/nature13258 24814341

[B55] Wyeth MS, Pelkey KA, Yuan X, Vargish G, Johnston AD, Hunt S, Fang C, Abebe D, Mahadevan V, Fisahn A, Salter MW, McInnes RR, Chittajallu R, McBain CJ (2017) Neto auxiliary subunits regulate interneuron somatodendritic and presynaptic kainate receptors to control network inhibition. Cell Rep 20:2156–2168. 10.1016/j.celrep.2017.08.017 28854365 PMC5600503

[B56] Zhang W, St-Gelais F, Grabner CP, Trinidad JC, Sumioka A, Morimoto-Tomita M, Kim KS, Straub C, Burlingame AL, Howe JR, Tomita S (2009) A transmembrane accessory subunit that modulates kainate-type glutamate receptors. Neuron 61:385–396. 10.1016/j.neuron.2008.12.014 19217376 PMC2803770

